# Nonlinear Errors Resulting from Ghost Reflection and Its Coupling with Optical Mixing in Heterodyne Laser Interferometers

**DOI:** 10.3390/s18030758

**Published:** 2018-03-02

**Authors:** Haijin Fu, Yue Wang, Pengcheng Hu, Jiubin Tan, Zhigang Fan

**Affiliations:** 1 Institute of Ultra-Precision Optoelectronic Instrument Engineering, Harbin Institute of Technology, Harbin 150001, China; haijinfu@hit.edu.cn (H.F.); wy206@stu.hit.edu.cn (Y.W.); jbtan@hit.edu.cn (J.T.); 2 Postdoctoral Research Station of Optical Engineering, Harbin Institute of Technology, Harbin 150001, China; fzg@hit.edu.cn

**Keywords:** laser sensor, heterodyne interferometer, optical nonlinearity, ghost reflection, optical mixing

## Abstract

Even after the Heydemann correction, residual nonlinear errors, ranging from hundreds of picometers to several nanometers, are still found in heterodyne laser interferometers. This is a crucial factor impeding the realization of picometer level metrology, but its source and mechanism have barely been investigated. To study this problem, a novel nonlinear model based on optical mixing and coupling with ghost reflection is proposed and then verified by experiments. After intense investigation of this new model’s influence, results indicate that new additional high-order and negative-order nonlinear harmonics, arising from ghost reflection and its coupling with optical mixing, have only a negligible contribution to the overall nonlinear error. In real applications, any effect on the Lissajous trajectory might be invisible due to the small ghost reflectance. However, even a tiny ghost reflection can significantly worsen the effectiveness of the Heydemann correction, or even make this correction completely ineffective, i.e., compensation makes the error larger rather than smaller. Moreover, the residual nonlinear error after correction is dominated only by ghost reflectance.

## 1. Introduction

Picometer level metrology faces increasing demand in numerous key fields, such as gravitational-wave detection, semiconductor industry, and nanotechnology [1,2,3,4,5]. In the case of Laser Interferometer Space Antenna (LISA) for detecting gravitational waves, pm-sensitivity is required to measure the transition of the inertial proof mass [1]. For precision positioning in semiconductor manufacturing, an uncertainty of about 25 pm should be available in the next decade according to the International Technology Roadmap for Semiconductors (ITRS) [2,3]. To deal with the challenges raised by the rapid progress of nanotechnology, the NANOTRACE project, funded by the European Metrology Research Programme (EMRP), has spearheaded the development of the next generation of optical interferometers with a target uncertainty of 10 pm [4].

Heterodyne laser interferometry is considered a promising candidate for the next generation of optical interferometer due to its high accuracy and robust capabilities. However, it is often subject to periodic nonlinear errors induced by optical mixing, with an amplitude averaging from several to tens of nanometers. Over the years, various solutions to this problem have been proposed, which fall into two overarching categories. The first adopts optical configurations with two spatially separated beams [6,7,8,9,10] and thereby avoids the optical mixing, but at the cost of complicated structures that are sensitive to air disturbance and thermal drift. Additionally, recent research indicates that these configurations still suffer from nonlinearities caused by multi-order Doppler frequency shift (DFS) arising from ghost reflection [11]. The second solution keeps the common optical paths and employs compensation algorithms, mainly the Heydemann correction [12,13,14,15,16,17]. This correction relies on the traditional model that first- and second-order nonlinearities are generated from optical mixing [18,19,20]. In theory, the correction is capable of eliminating nonlinearities through elliptical fitting. Nevertheless, residual nonlinear errors from hundreds of picometers to several nanometers are still found even after compensation [12,13,14,15,16,17], the cause and mechanism of which remain largely uninvestigated.

In this paper, to study the residual nonlinear errors impeding the realization of picometer level metrology, a novel nonlinearity model based on ghost reflection and its coupling with optical mixing is proposed and then verified by experiments. Further, the influence of the developed model on the Heydemann correction is analyzed to reveal the source and mechanism of the residual nonlinear errors after compensation.

## 2. Nonlinearity Model Based on Ghost Reflection and Its Coupling with Optical Mixing

**Figure 1:**
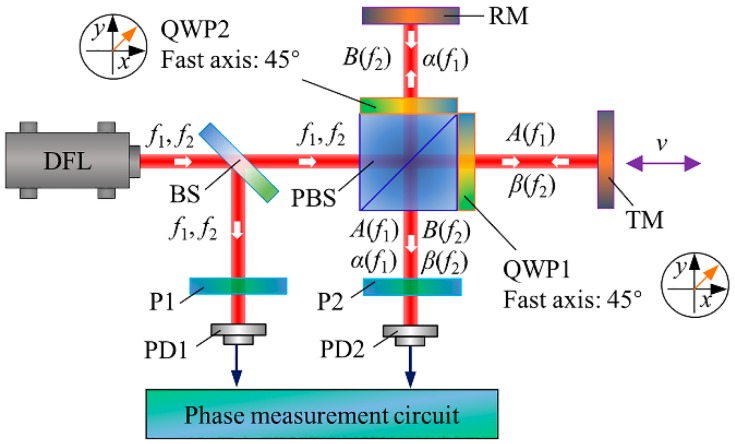
Schematic of a typical heterodyne interferometer. DFL: dual-frequency laser. BS: beam splitter. PBS: polarizing beam splitter. RM: reference mirror. TM: target mirror. QWP: quarter wave-plate. P: Polarizer. PD1: photodetector for the reference signal. PD2: photodetector for the measurement signal.

The schematic of a typical heterodyne interferometer is shown in Figure 1. The laser output, consisting of two orthogonal polarized beams with slightly different frequencies of *f*_1_ and *f*_2_, is split in energy by a beam splitter (BS) into two parts: one is detected by the photodetector 1 (PD1) and serves as the reference signal *I*_r_; the other, in an ideal case, is completely separated in polarization by a polarizing beam splitter (PBS) into measurement and reference beams with frequencies of *f*_1_ and *f*_2_, respectively. The two beams are directed to the movable and fixed mirrors (TM and RM) in the measurement and reference arms, respectively. The reflected beams are recombined and then detected by PD2 as the measurement signal *I*_m_. The quarter-wave-plates (QWP1 and QWP2) positioned in the measurement and reference paths are used to rotate the beam polarization orientation by 90°, and thereby a convenient layout with the input and output beams located on two adjacent sides of the PBS can be realized. The measurement of phase difference between the measurement and reference signals, which is proportional to the displacement of the target mirror, is carried out electronically.

Optical mixing is inevitable in a real case due to the ellipticity and nonorthogonality of the laser source, optical defects, and alignment errors. Therefore, each arm of the heterodyne interferometer contains a small component of the frequency intended for the other arm. In this case, the laser beams back from the measurement and reference arms are given by

(1) $$E_{m}=A\exp\left[i\left(\omega_{1}t+\phi \right)\right]+\beta \exp\left[i\left(\omega_{2}t+\phi \right)\right],$$

(2) $$E_{r}=B\exp\left(i\omega_{2}t\right)+\alpha \exp\left(i\omega_{1}t\right),$$

where *A* and *B* are amplitudes of the intended measurement and reference beams, respectively; *β* and *α* are amplitudes of the unwanted leakage laser beams in the measurement and reference arms, respectively. *ω*_1_ = 2*πf*_1_ and *ω*_2_ = 2*πf*_2_ are angular frequencies of the laser beams, ϕ=4πL/λ=4πvt/λ denotes the phase shift resulting from DFS which is equivalent to that caused by target displacement. For simplicity, initial phases of the laser beams are set at zero. 

**Figure 2:**
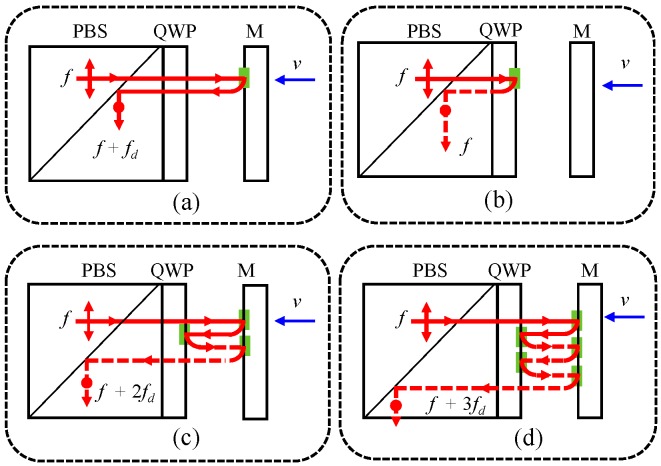
Presentation of ghost beams in the measurement arm of a heterodyne laser interferometer. (**a**) Intended beam with first-order Doppler frequency shift (DFS); (**b**) Ghost beam with zeroth-order DFS; (**c**) Ghost beam with second-order DFS; (**d**) Ghost beam with third-order DFS.

Besides optical mixing, ghost reflection that occurs at the interface of optical components in practical applications can also lead to nonlinear errors by inducing multi-order DFS [11]. This is a potential obstacle of picometer level metrology as the existing compensation algorithms rely on a traditional nonlinearity model based on optical mixing. As illustrated in Figure 2a–c, the intended measurement beam is subject to the first-order DFS caused by target motion, while ghost reflections in the inner and outer surfaces of the QWP result in the zeroth- and second-order DFS, respectively. In Figure 2d, ghost reflection happens twice in the outer surface of the QWP, leading to a third-order DFS. Similarly, the unwanted leakage laser beam in the measurement arm is also subject to multi-order DFS, and it will participate in the inference that produces the measurement signal. In this case, the beam back from the measurement arm is rewritten as

(3) $$E_{m}=A\left\{\sum_{k=0}^{n}\gamma_{k}\exp\left[i\left(\omega_{1}t+k\phi \right)\right]\right\}+\beta \left\{\sum_{k=0}^{n}\gamma_{k}\exp\left[i\left(\omega_{2}t+k\phi \right)\right]\right\}，$$

where

(4) $$\gamma_{k}=\left\{ \begin{array}{ll} r & k=0\\ {\left(1-r\right)}^{2}r^{k-1} & k\geq 1 \end{array}\right.,$$

*k* represents the order of DFS and *r* denotes the ghost reflectance.

The optical components in a laser interferometer are generally coated with anti-reflection films which make the ghost reflectance quite small. Therefore, when evaluating the measurement signal, a third-order approximation is sufficient, i.e., only the zero-, first-, second- and third-order DFS are necessary for computation, with any other high-order DFS omitted. Regarding any beams back from the reference arm, they are free of DFS and can still be expressed as Equation (2). Under the approximation, the measurement signal of the interferometer can be calculated as follows:

(5) Im=Re[(Em+Er)⋅(Em+Er)∗]=Γ0cos(Δωt+ϕ)︸base+Γ−1cos(Δωt+2ϕ)︸−1st harmonic+Γ−2cos(Δωt+3ϕ)︸−2nd harmonic+Γ1cos(Δωt)︸1st harmonic+Γ2cos(Δωt−ϕ)︸2nd harmonic+Γ3cos(Δωt−2ϕ)︸3rd harmonic+Γ4cos(Δωt−3ϕ)︸4th harmonic,

where ∆*ω* = *ω*_1_ − *ω*_2_ = 2*π*∆*f* and

(6) $$\left\{ \begin{array}{l} \Gamma_{0}=A\beta \gamma_{0}\gamma_{1}+A\beta \gamma_{1}\gamma_{2}+A\beta \gamma_{2}\gamma_{3}+AB\gamma_{1}\\ \Gamma_{-1}=A\beta \gamma_{0}\gamma_{2}+A\beta \gamma_{1}\gamma_{3}+AB\gamma_{2},\Gamma_{-2}=A\beta \gamma_{0}\gamma_{3}+AB\gamma_{3}\\ \Gamma_{1}=A\beta \gamma_{0}^{2}+A\beta \gamma_{1}^{2}+A\beta \gamma_{2}^{2}+A\beta \gamma_{3}^{2}+AB\gamma_{0}+\alpha \beta \gamma_{0}+B\alpha \\ \Gamma_{2}=A\beta \gamma_{0}\gamma_{1}+A\beta \gamma_{1}\gamma_{2}+A\beta \gamma_{2}\gamma_{3}+\alpha \beta \gamma_{1}\\ \Gamma_{3}=A\beta \gamma_{0}\gamma_{2}+A\beta \gamma_{1}\gamma_{3}+\alpha \beta \gamma_{2},\Gamma_{4}=A\beta \gamma_{0}\gamma_{3}+\alpha \beta \gamma_{3}. \end{array}\right.$$

**Figure 3:**
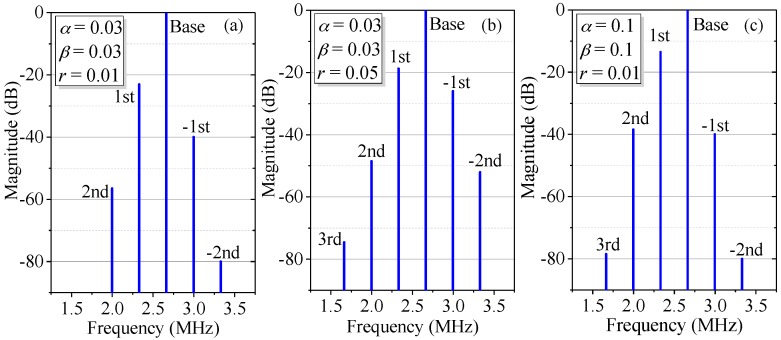
Simulation spectra of the measurement signal with different degrees of optical mixing and ghost reflectance, where *A* = *B* = 1, Δ*f* = 2.33 MHz, *v* = 105 mm/s, (**a**) with *α* = *β* = 0.03, *r* = 0.01; (**b**) with *α* = *β* = 0.03, *r* = 0.05; (**c**) with *α* = *β* = 0.1, *r* = 0.01.

In Equation (5), the first, fourth, and fifth terms are the base signal, the first- and second-order nonlinear harmonics, respectively. They are similar to those of the conventional model based on optical mixing, but with different magnitudes due to ghost reflection. The second and third terms, which arise from the second- and third-order DFS, are referred to as negative first- and negative second-order harmonics, respectively, for their phases are opposite in sign to that of the traditional first- and second-order harmonics. The sixth and seventh terms, which are the third- and fourth-order harmonics, are induced by the coupling of ghost reflection and optical mixing. Figure 3 shows the simulation spectra of the measurement signal with different degrees of optical mixing and ghost reflectance. For convenience, in this paper *A* and *B* are set at 1, whereas *α* and *β* represent the degree of optical mixing. When *α* = *β* = 0.03, *r* = 0.01, as illustrated by Figure 3a, besides the base signal, the traditional first- and second-order nonlinear harmonics, the negative first- and negative second-order nonlinear harmonics coming from multi-order DFS can also be observed, with magnitudes smaller relative to those of the traditional first- and second-order nonlinear harmonics, respectively; the third- and fourth-order harmonics, caused by coupling of ghost reflection and optical mixing, are too small to be seen. If *r* increases to 0.05 or *α* and *β* are enlarged to 0.1, as shown in Figure 3b,c, the third-order nonlinear harmonic can be observed due to the enhancement of coupling of ghost reflection and optical mixing; however, the fourth-order nonlinear harmonic still cannot be seen. In addition, compared to Figure 3a, the apparent growth of the first-, negative first-, second- and negative second-order harmonics in Figure 3b can be ascribed to an increase of the ghost reflectance; in Figure 3c, the increase of the leakage beams leads to growth in the traditional first- and second-order harmonics.

Similar to the traditional nonlinear harmonics, the new harmonics arising from ghost reflection and its coupling with optical mixing will also contribute to the nonlinearity of an interferometer. According to Equation (5), the overall nonlinear phase error can be calculated by using the phasor analysis method [21] which is given as follows:

(7) $$\delta \varphi =\arctan\left(\frac{\sum_{n=-2,\;n\neq 0}^{n=4}\Gamma_{n}\sin(n\phi )}{\sum_{n=-2}^{n=4}\Gamma_{n}\cos\left(n\phi \right)}\right).$$

In real applications, the magnitudes of the leakage laser beams are significantly smaller than those of the intended beams and the ghost reflection is comparably slight, which makes *Γ_n_* ≪ *Γ*_0_, *n* ≠ 0. Accordingly, Equation (7) can be further simplified as follows:

(8) $$\delta \varphi \approx \frac{\Gamma_{1}-\Gamma_{-1}}{\Gamma_{0}}\sin(\phi )+\frac{\Gamma_{2}-\Gamma_{-2}}{\Gamma_{0}}\sin(2\phi )+\frac{\Gamma_{3}}{\Gamma_{0}}\sin\left(3\phi \right)+\frac{\Gamma_{4}}{\Gamma_{0}}\sin(4\phi ).$$

It can be seen from Equation (8) that the negative first- and negative second-order harmonics arising from ghost reflection result in the negative first- and negative second-order nonlinear errors, opposite in sign to the traditional first- and second-order nonlinearities. Additionally, the third- and fourth-order harmonics, which are induced by the coupling of ghost reflection and optical mixing, lead to completely new nonlinearities.

## 3. Experimental Validation

**Figure 4:**
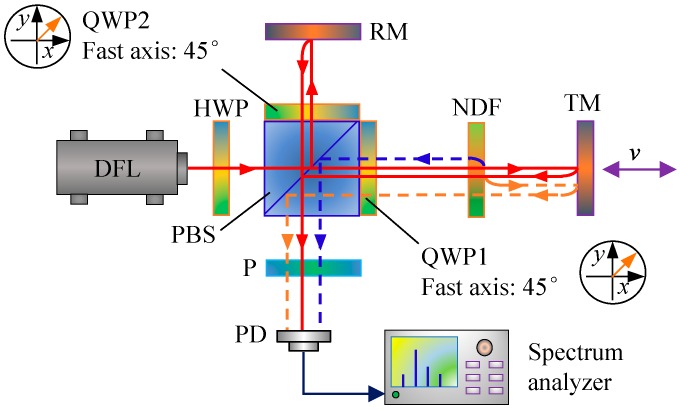
Experimental interferometer setup used for validation. DFL: dual-frequency laser. BS: beam splitter. PBS: polarizing beam splitter. RM: reference mirror. TM: target mirror. QWP: quarter wave-plate. P: Polarizer. PD: photodetector. HWP: half wave-plate. NDF: neutral density filter.

To verify the developed model, as shown in Figure 4, an experimental setup of a typical interferometer was established, where the output laser of a commercial laser source (Agilent 5517B, Agilent Technologies, Santa Clara, CA, USA) consisted of two orthogonal linear polarized beams with a frequency difference of 2.33 MHz at the central wavelength of 632.8 nm. A half wave-plate (HWP) was employed to rotate the beam polarization orientation and thereby to adjust the amplitudes of leakage beams. To simulate variable ghost reflectance, a reflective neutral density filter (NDF) with a continuously tunable reflectance was positioned between a target mirror and QWP1. The target mirror was mounted on a linear motorized translation stage. A PIN photodetector (HCA-S-200M-SI, FEMTO Messtechnik GmbH, Berlin, Germany) was adopted to conduct photoelectric conversion of the measurement signal which was then analyzed by a spectrum analyzer (Agilent N9010A, Agilent Technologies, Santa Clara, CA, USA).

**Figure 5:**
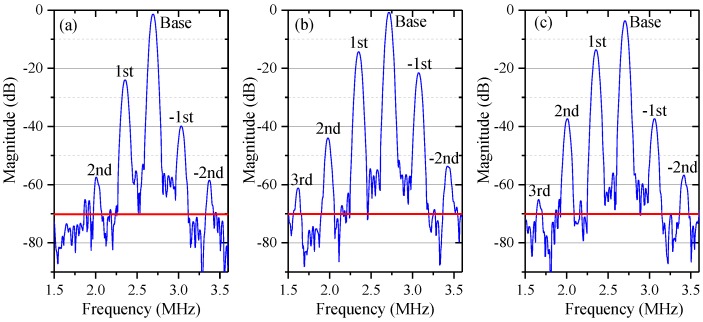
Experimental spectra of the measurement signal. (**a**) Without HWP and NDF; (**b**) Adopting NDF to enhance the ghost reflectance; (**c**) Adopting HWP to increase the leakage beams. The red line in each panel denotes a background noise level of −70 dB.

Figure 5 shows the experimental spectra of the measurement signal with different degrees of optical mixing and ghost reflectance. The red horizontal line in each panel denotes a background noise level of −70 dB. Nonlinear harmonics below this line will not appear in real spectra. In Figure 5a (without the HWP and NDF), the leakage beams and the ghost reflectance are relatively small. The third- and fourth-order nonlinear harmonics, which are theoretically generated by the coupling of ghost reflection and optical mixing, could not be identified. When enlarging the leakage beams by rotating the HWP or increasing the ghost reflectance by adjusting the NDF, as shown in Figure 5b, the third-order nonlinear harmonic broke through the background noise and presented in the spectra, as illustrated in Figure 5b,c, but the fourth-order nonlinear harmonic was still submerged below the red line. In addition, compared to Figure 5a, the enlarged ghost reflectance in Figure 5b also led to evident growth of the first-, negative first-, second- and negative second-order harmonics, which is largely consistent with the variation trend between Figure 3a and Figure 3b. In Figure 5c, the increased leakage beams also resulted in distinct growth of the traditional first- and second-order harmonics, but their influence on the negative first- and negative second-order harmonics was much lower, which corresponds with the variation trend between Figure 3a,c.

## 4. Influence of the Proposed Nonlinearity Model on the Heydemann Correction

In real applications, the leakage beams and the ghost reflectance cannot be very large. This means that the nonlinear harmonics induced by ghost reflection and its coupling with optical mixing are generally relatively smaller than the traditional nonlinear harmonics and accordingly might contribute little to the overall nonlinear error. However, if we want to achieve sub-nanometer or picometer accuracy and simultaneously take advantage of the interferometers with common paths, such as relatively simple configuration and higher thermal stability, the periodic error in this kind of interferometers must be addressed. One well-known method is the Heydemann correction algorithm [12]. To apply this method to heterodyne laser interferometers, one needs to transform the measurement and reference signals into orthogonal form [13]. More specifically, the measurement signal needs to be mixed with two reference signals 90° out of phase, i.e., *I*_m_ ⨂ *I*_r_ (0°) and *I*_m_ ⨂ *I*_r_ (90°). After a low-pass filter, two slow varying direct current (DC) terms can be obtained. For the setup in Figure 1, the measurement signal *I*_m_ can be calculated by Equation (5) and the reference signal *I*_r_ can be simplified as cos (∆*ωt*). Therefore, the slowly varying DC terms can be derived as follows:

(9) $$\left\{ \begin{array}{l} I_{x}=\frac{\Gamma_{1}}{2}+\frac{\Gamma_{0}+\Gamma_{2}}{2}\cos\left(\phi \right)+\underset{\mathrm{DF}\;\mathrm{term}}{\underset{︸}{\frac{\Gamma_{-1}+\Gamma_{3}}{2}\cos\left(2\phi \right)}}+\underset{\mathrm{TF}\;\mathrm{term}}{\underset{︸}{\frac{\Gamma_{-2}+\Gamma_{4}}{2}\cos\left(3\phi \right)}}\\ I_{y}=\frac{\Gamma_{2}-\Gamma_{0}}{2}\sin\left(\phi \right)+\underset{\mathrm{DF}\;\mathrm{term}}{\underset{︸}{\frac{\Gamma_{3}-\Gamma_{-1}}{2}\sin\left(2\phi \right)}}+\underset{\mathrm{TF}\;\mathrm{term}}{\underset{︸}{\frac{\Gamma_{4}-\Gamma_{-2}}{2}\sin\left(3\phi \right)}} \end{array}\right..$$

**Figure 6:**
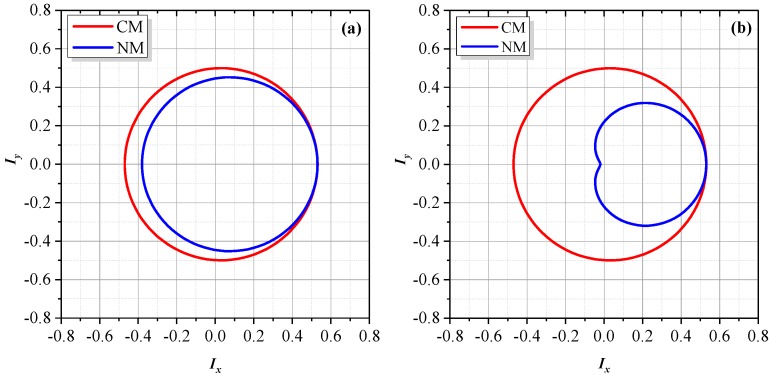
The Lissajous trajectories for the conventional model (CM) and the proposed new model (NM), with *α* = *β* = 0.03, *r* = 0.05. (**a**) The difference between the two trajectories is amplified by a factor of 1. (**b**) The difference between the two trajectories is amplified by a factor of 5.

The high-order terms in Equation (9), including the DF (double frequency) and TF (triple frequency) terms, come from the new nonlinear harmonics in the proposed nonlinearity model, which are induced by ghost reflection and its coupling with optical mixing. Figure 6a illustrates the Lissajous trajectory between *I_y_* and *I_x_*, with *α* = *β* = 0.03, *r* = 0.05. For comparison, the graph based on the conventional model is also presented. For the conventional model, those high-order terms in Equation (9) are zero, thus the trajectory of *I_y_* versus *I_x_* is a perfect ellipse. As for the proposed model, the Lissajous trajectory still looks like an ellipse and the major visible changes are at the center as well as the major and minor axes. However, if the difference between the two trajectories in Figure 6a is amplified by a factor of 5, as shown in Figure 6b, for the proposed model, the Lissajous trajectory looks like a cardioid. This can be interpreted by Equation (9). Regarding the high-order terms, the amplitudes of the DF terms are significantly larger than those of the TF terms, thus distortion is mainly caused by the DF terms. If the TF terms are neglected, Equation (9) becomes a cardioid equation. In real applications, as the ghost reflectance is generally quite small, the distortion might not be identified easily.

**Figure 7:**
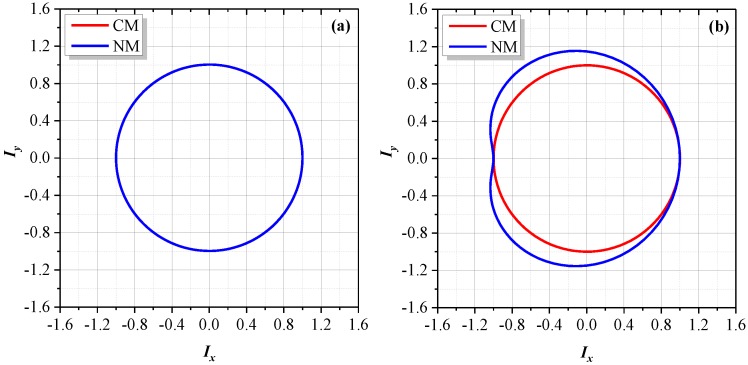
The corrected Lissajous trajectories for the conventional model (CM) and the proposed new model (NM), with *α* = *β* = 0.03, *r* = 0.05. (**a**) The difference between the two trajectories is amplified by a factor of 1. (**b**) The difference between the two trajectories is amplified by a factor of 7.

Figure 7a shows the Lissajous trajectories after Heydemann correction for the conventional model. In theory, the nonlinear errors can be completely corrected through elliptical fitting, making the corrected trajectory a perfect circle centering at the origin of the coordinates. For the proposed model, the corrected trajectory almost overlaps with that of the conventional model. As shown in Figure 7a, there is no distinguishable deviation. If the difference between these two corrected trajectories is amplified by a factor of 7, as illustrated in Figure 7b, the deviation can be identified. For the proposed model, the corrected graph is still a cardioid. However, in a real case, this deviation might be invisible due to the generally quite small ghost reflection.

**Figure 8:**
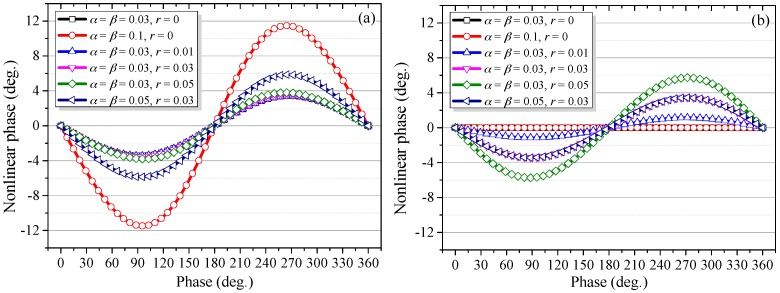
Nonlinear phases with different degrees of optical mixing and ghost reflectance, (**a**) before Heydemann correction; (**b**) after Heydemann correction.

In order to quantify the influence of the high-order nonlinear harmonics, the nonlinear phases before and after the Heydemann correction were calculated. Results are provided in Figure 8. It can be seen from Figure 8a, for the several given cases, that the periodic errors are dominated by the first-order nonlinearity. When the amplitudes of leakage beams are kept at *α* = *β* = 0.03 and ghost reflectance is set at 0, 0.01, 0.03, and 0.05, respectively, the nonlinearities for these cases are almost overlapped, indicating that ghost reflection generally contributes little to the overall error. However, if *α* and *β* increase from 0.03 to 0.05 and 0.1, there are significant increases of the nonlinearities. Therefore, the magnitudes of the nonlinearities are determined predominantly by the amplitudes of leakage beams. Figure 8b shows the nonlinear phases after the Heydemann correction, when the ghost reflection is zero, such as the cases *α* = *β* = 0.03, *r* = 0 and *α* = *β* = 0.1, *r* = 0; the nonlinear errors can be fully corrected regardless of the magnitudes of leakage beams. When *r* = 0, the proposed nonlinearity model is identical to the traditional model. Otherwise, these errors cannot be effectively compensated even with a tiny ghost reflectance of *r* = 0.01. Moreover, the residual error increases sharply with increased ghost reflectance. For the case *α* = *β* = 0.03, *r* = 0.01, the residual error is smaller than that before correction. For the two cases with the same ghost reflectance, i.e., *α* = *β* = 0.03, *r* = 0.03 and *α* = *β* = 0.05, *r* = 0.03, the residual errors are almost the same. This suggests that the residual errors are determined only by the ghost reflectance and are irrelevant to the leakage beams. Additionally, the magnitude of the residual error for the case *α* = *β* = 0.03, *r* = 0.03 is close to that before correction. In contrast, for the case *α* = *β* = 0.03, *r* = 0.05, the residual error is even significantly larger than that before correction. Therefore, based on the above analysis, though ghost reflection and its coupling with optical mixing has a negligible influence on the overall nonlinearity, it can significantly reduce the effectiveness of the Heydemann correction or even make this compensation algorithm completely ineffective.

## 5. Mechanism of the Residual Nonlinear Errors after the Heydemann Correction

As described above, an “abnormality” is a case in which the Heydemann correction makes nonlinear errors larger. For instance, when *α* = *β* = 0.03 and *r* = 0.05, as in Figure 7a, there is no visible deviation in the corrected Lissajous trajectory when compared to the conventional model. In contrast, in Figure 8b, the compensated phase is higher than that before correction. The Heydemann correction is an ellipse-fitting algorithm based on the least square method. First, it fits the Lissajous trajectory to obtain the ellipse parameters, including the center of the ellipse *x*_0_ and *y*_0_, the semimajor and semiminor axes of the ellipse *a* and *b*, and the non-orthogonal angle *φ*_0_ between the signals *I_x_* and *I_y_*. Next, these parameters are used to correct the original signals and thereby remove the nonlinear errors. Finally, the real measurement phase can be obtained with the corrected signals by arctangent operation. In order to analyze the above-mentioned abnormality for the Lissajous trajectory based on the proposed model, a series of ellipse-fittings were carried out and the fitted parameters are listed in Table 1. The amplitudes of the nonlinear harmonics Γ*_n_* (*n* = −2, −1, 0, 1, 2) are also presented in the table.

According to Table 1, it can be found that the ellipse-fitting parameters and the amplitudes of the nonlinear harmonics meet the relation as follows:

(10) $$x_{0}\approx \frac{\Gamma_{1}+\Gamma_{-1}}{2},\;y_{0}=0,\;a\approx \frac{\Gamma_{0}+\Gamma_{-2}+\Gamma_{2}}{2},\;b\approx \frac{\Gamma_{0}+\Gamma_{-2}-\Gamma_{2}}{2}.$$

Generally, Γ_2_, Γ_−2_ ≪ Γ_0_, thus Equation (10) can be further rewritten as

(11) $$x_{0}=\frac{\Gamma_{1}+\Gamma_{-1}}{2},\;y_{0}=0,\;a\approx b\approx \frac{\Gamma_{0}}{2}.$$

In this approximation, the “corrected signals” of Equation (9) can be obtained as

(12) $$\left\{ \begin{array}{l} I_{x}^{\prime }=\frac{I_{x}-x_{0}}{a}=\frac{1}{a}\left[-\frac{\Gamma_{-1}}{2}+\frac{\Gamma_{0}+\Gamma_{2}}{2}\cos\left(\phi \right)+\underset{\mathrm{DF}\;\mathrm{term}}{\underset{︸}{\frac{\Gamma_{-1}+\Gamma_{3}}{2}\cos\left(2\phi \right)}}+\underset{\mathrm{TF}\;\mathrm{term}}{\underset{︸}{\frac{\Gamma_{-2}+\Gamma_{4}}{2}\cos\left(3\phi \right)}}\right]\\ I_{y}^{\prime }=\frac{I_{y}-y_{0}}{b}\approx \frac{1}{a}\left[\frac{\Gamma_{2}-\Gamma_{0}}{2}\sin\left(\phi \right)+\underset{\mathrm{DF}\;\mathrm{term}}{\underset{︸}{\frac{\Gamma_{3}-\Gamma_{-1}}{2}\sin\left(2\phi \right)}}+\underset{\mathrm{TF}\;\mathrm{term}}{\underset{︸}{\frac{\Gamma_{4}-\Gamma_{-2}}{2}\sin\left(3\phi \right)}}\right]. \end{array}\right.$$

**Figure 9:**
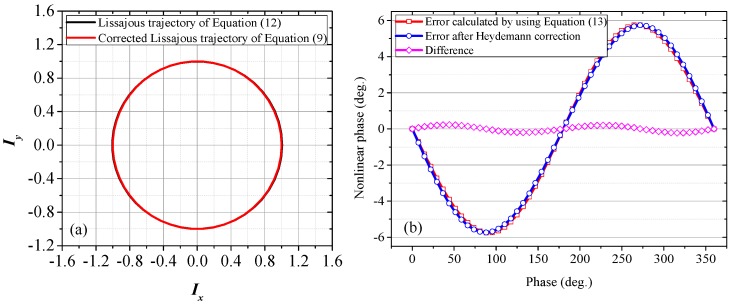
(**a**) Lissajous trajectories calculated by using Equation (12) and corrected Lissajous trajectory of Equation (9); (**b**) Nonlinear errors calculated by using Equation (13) and residual error obtained by directly applying the Heydemann correction to Equation (9). *A* = *B* = 1, *α* = *β* = 0.03, *r* = 0.05.

As Equation (12) is an approximate formula, to verify its reliability, when *α* = *β* = 0.03, *r* = 0.05, the Lissajous trajectory calculated by using Equation (12) is compared with the corrected Lissajous trajectory of Equation (9), which is obtained by directly applying the Heydemann correction to Equation (9). As illustrated in Figure 9a, these two trajectories almost overlap, indicating a good approximation. If the TF terms in Equation (12) are omitted, Equation (12) will be a cardioid equation, which is the source of the heart-shaped distorted trajectory in Figure 7b. With reference to the relationship between Equations (8) and (9), the residual nonlinear phase error in Equation (12) can be backward derived as

(13) $$\delta \varphi^{\prime }\approx -\frac{2\Gamma_{-1}}{\Gamma_{0}}\sin\left(\phi \right)+\frac{\Gamma_{2}-\Gamma_{-2}}{\Gamma_{0}}\sin(2\phi )+\frac{\Gamma_{3}}{\Gamma_{0}}\sin\left(3\phi \right)+\frac{\Gamma_{4}}{\Gamma_{0}}\sin(4\phi ).$$

When *α* = *β* = 0.03, *r* = 0.05, the nonlinear error calculated by using Equation (13) is shown in Figure 9b. For comparison, the residual error obtained by directly applying the Heydemann correction to Equation (9) is also illustrated in the same figure. The two curves are basically consistent and the small difference can be attributed largely to the approximation of *a* ≈ *b*. For the proposed model, Equation (13) can be used to estimate the residual phase after the Heydemann correction.

As illustrated in Figure 8, for the proposed model, if the ghost reflectance is not zero, whether the Heydemann correction is applied or not, the first-order nonlinearity dominates the overall nonlinear error. Generally, the ghost reflectance is a quite small value, according to Equations (4)–(6) and (8). Before correction, the amplitude of the first-order nonlinearity can be evaluated by

(14) $$\frac{\left(\Gamma_{1}-\Gamma_{-1}\right)}{\Gamma_{0}}\approx \frac{A\beta +B\alpha }{AB}=\alpha +\beta .$$

For simplicity, in this paper, the values *A* and *B* are always set at 1. Accordingly, before the Heydemann correction, the first-order nonlinearity is determined mainly by the optical mixing degree. Similarly, according to Equation (13), after correction, the amplitude of the first-order nonlinearity can be evaluated by

(15) $$\frac{2\Gamma_{-1}}{\Gamma_{0}}\approx 2r,$$

which is only determined by the ghost reflectance. With the help of Equations (14) and (15), the above-mentioned abnormality in Figure 8 can be reasonably interpreted. For case *α* = *β* = 0.03, *r* = 0 and *α* = *β* = 0.1, *r* = 0, as the ghost reflectance is zero, there is no residual error after correction. For case *α* = *β* = 0.03, *r* = 0.01, 2*r* < α + β, the residual error decreases after correction. For case *α* = *β* = 0.03, *r* = 0.03, because 2*r* = *α* + *β*, no clear difference can be observed between the nonlinear errors before and after correction. For case *α* = *β* = 0.05, *r* = 0.03, as 2*r* < *α* + *β*, the residual error decreases after correction, but is very close to case *α* = *β* = 0.03, *r* = 0.03, due to the same ghost reflectance. By contrast, in case *α* = *β* = 0.03, *r* = 0.05, then 2*r* > *α* + *β*, compensation leads to a larger error instead.

## 6. Conclusions

Even after the Heydemann correction, residual nonlinear errors, ranging from hundreds of picometers to several nanometers, are still found in heterodyne laser interferometers. This is a crucial factor impeding the realization of picometer level metrology, but its source and mechanism have barely been investigated. To study this problem, a novel nonlinear model based on optical mixing and coupling with ghost reflection is proposed and then verified by experiments. After intense investigation of this new model’s influence, results indicate that the new additional high-order and negative-order nonlinear harmonics, arising from ghost reflection and its coupling with optical mixing, have only a negligible contribution to the overall nonlinear error. In real applications, any effect on the Lissajous trajectory might be invisible due to the small ghost reflectance. However, even a tiny ghost reflection can significantly worsen the effectiveness of the Heydemann correction, or even make this correction completely ineffective, i.e., compensation makes the error larger rather than smaller. Moreover, the residual nonlinear error after correction is dominated only by the ghost reflectance. Therefore, for real applications that intend to achieve sub-nanometer or picometer level accuracy, ghost reflection and its coupling with optical mixing should be taken into serious consideration, and the design must be elaborated to restrict ghost reflection. This study is expected to promote the development of ultra-high precision laser interferometry.

## Figures and Tables

**Table 1:** Ellipse-fitting parameters with different degrees of optical mixing and ghost reflectance.

***α***	**0.03**	**0.1**	**0.03**	**0.05**	**0.03**	**0**	**0**
***β***	0.03	0.1	0.03	0.05	0.03	0	0
***r***	0	0	0.03	0.03	0.05	0.05	0.03
**Г_1_**	0.06	0.2	0.0866	0.1244	0.1046	0.05	0.03
**Г_−1_**	0	0	0.0283	0.0283	0.0453	0.0451	0.0282
**Г_2_**	9 ${\times \;10}^{-4}$	0.01	0.0025	0.0051	0.0034	0	0
**Г_−2_**	0	0	8.5 ${\times \;10}^{-4}$	8.5${\;\times \;10}^{-4}$	0.0023	0.0023	8.5${\;\times \;10}^{-4}$
**Г_0_**	1	1	0.9425	0.9436	0.9051	0.9025	0.9409
***x*_0_**	0.03	0.1	0.0575	0.0765	0.0751	0.0476	0.0291
***y*_0_**	0	0	0	0	0	0	0
***a***	0.5005	0.5050	0.4729	0.4748	0.4554	0.4524	0.4709
***b***	0.4996	0.4950	0.4705	0.4697	0.4520	0.4524	0.4709
$\varphi_{0}$	0	0	0	0	0	0	0
